# Rare chondroblastoma of the 6th left rib, video-assisted thoracoscopy resected: one case report and literature review

**DOI:** 10.1186/s13019-021-01572-1

**Published:** 2021-07-07

**Authors:** Yonghui Wu, Jiexia Guan, Kai Zhang, Huiguo Chen, Weibin Wu, Jian Zhang

**Affiliations:** 1 Department of Cardiothoracic Surgery, the Third Affiliated Hospital of Sun Yat-Sen University, No.600 Tianhe Road, Guangzhou, China; 2 Department of Pathology, The Third Affiliated Hospital of Sun Yat-Sen University, No. 600 Tianhe Road, Guangzhou, China

**Keywords:** Rib tumor, Chondroblastoma, Aggressive tumor, VATS, Case report

## Abstract

**Introduction:**

Chondroblastoma is a rare, benign locally but aggressive bone tumor. It accounts for < 1% of primary bony tumors, and mostly arises from long bones; the rib chondroblastoma is especial rare. Due to its rarity, there are no definitive or standard treatment guidelines.

**Case presentation:**

A case of a 24-year-old male with a chondroblastoma located on the 6th posterior left rib. Computed tomography (CT) demonstrated a rib tumor that was a well-defined oval lesion of 20 mm × 18 mm, with lytic bone destruction. The imaging first diagnosis was Langerhans cell histiocytosis (LCH), a giant cell tumor, or other type of neoplasm. The whole tumor and a part of partial rib were resected by video-assisted thoracoscopy surgery (VATS). Pathological and immunohistochemical (IHC) examination made a diagnosis of chondroblastoma. Compared with traditional open thoracic surgery, VATS can achieve the same effects and cause less injury to patient. No postoperative adjuvant therapy was given, and had followed up 23 months after surgery, there was no recurrence or metastasis.

**Conclusion:**

Chondroblastoma has a risk of recurrence and metastasis, surgery plays an important role in the treatment of chondroblastoma, VATS can achieve the same outcome as traditional open thoracic surgery with less pain and lung function. Close follow-up is needed postoperative.

## Background

Chondroblastoma was first described as a “giant cell tumor with calcifications” by Kolodny in 1927, Ewing described it as a “calcifying giant cell tumor” in 1928, and Codman described it as an “epiphyseal chondromatous giant cell tumor” in 1931 [1]. Chondroblastoma is a benign locally but aggressive bone tumor that accounts for < 1% of primary bony tumors [2] . It characteristically occurs in the epiphyses and secondary ossification centers of long bones, particularly the femur and tibia. The chondroblastoma of the rib is very rare.

The World Health Organization (WHO) has defined chondroblastoma as originating in the epiphyses of skeletally immature persons [3]. The tumor can affect people of all ages, but usually occurs in adolescents and young adults, and men are more commonly affected than women with a male to female ratio of 2:1 [3–5]. About 40% of tumors occur in adolescents. Chondroblastoma is associated with recurrence and pulmonary metastases [6, 7]. Herein, we present the case of a 24-year-old male with a chondroblastoma of the 6th left rib that was removed by VATS. Following 23 months after surgery, the patient has no evidences of recurrence or metastasis.

## Case presentation

**Fig. 1:**
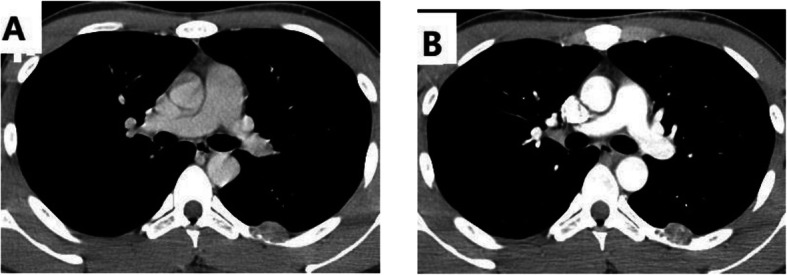
Chest CT showed that there was a tumor located the 6th posterior left rib and bony cortex erosion (A). Enhanced CT could saw the tumor non-uniformity enhancement (B)

A 24-year-old male was complained of intermediate dry cough for 1 month. He denied any history of fever, weight loss, chest pain. Chest CT showed a tumor located on the posterior 6th left rib, which measured about 20 mm × 18 mm. An area of local bony destruction with conserved cortex was noted, and the margins were delineated with some ossifying matrix. Enhanced CT revealed non-uniform enhancement. There was no evidence of extension to the adjacent soft tissue. The imaging first diagnosis was LCH, a giant cell tumor, or other type of neoplasm (Fig. 1).

His family medical history was negative for any type of malignancy. Physical examination, the tumor wasn’t palpable and no pain by pressing on the chest wall. The overlying skin was normal, and no sensory deficit. Laboratory testing results, including tumor markers, complete blood cell count and erythrocyte sedimentation rate (ESR) were within the normal value. The derivation purified protein skin test and tuberculosis test were negative too.

**Fig. 2:**
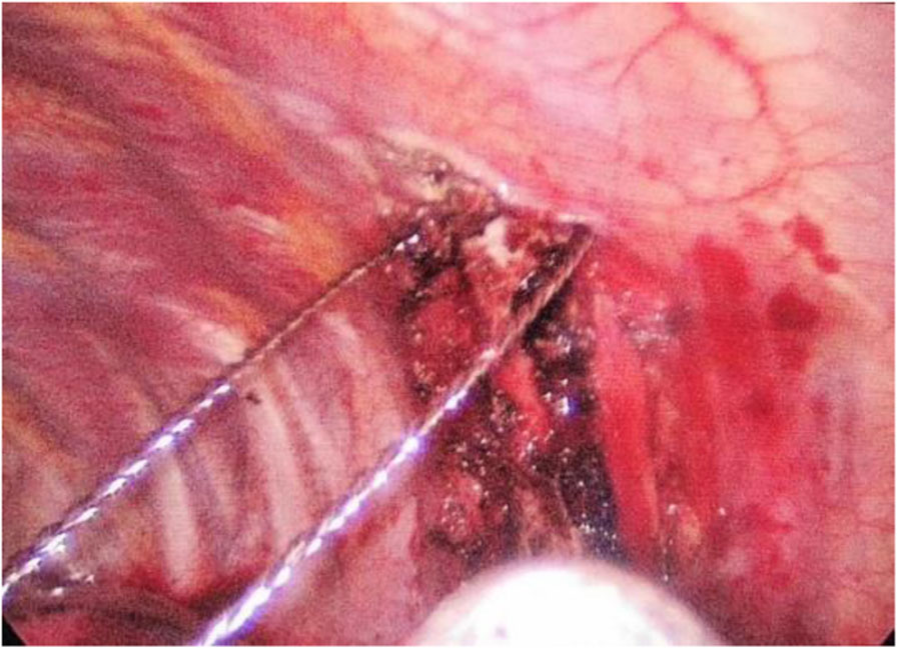
VATS showed that a thin and broken periosteal shell was exposed, the surrounding soft tissue wasn’t invaded by tumor. Removed the tumor and broken rib with wire saw

The patient was anesthetized with double-lumen endotracheal intubation and lied on the right side. The endoscopic incision about 1 cm length was the 8th intercostal space of the left posterior axillary line, and another operating incision about 3 cm length is the 4th intercostal anterior axillary line. The right lung was ventilated and the left lung was collapsed. It was observed to be present on the posterior side of the 6th left rib, the margins were well-defined, and no sign of adjacent soft tissue invasion was noted. The electrocoagulation hook kept about 0.5 cm away from the tumor border and completely resected the tumor. Frozen section pathological examination demonstrated a benign or a low-grade malignant bone tumor. The more surrounding tissues and partial intercostal muscle were removed further. The straight pliers were used to guide the ribs to separate the ribs, and the wire saws cut off the broken ribs, including a margin of at least 1 cm of normal rib. No reconstruction was required for the rib deficit (Fig. 2).

**Fig. 3:**
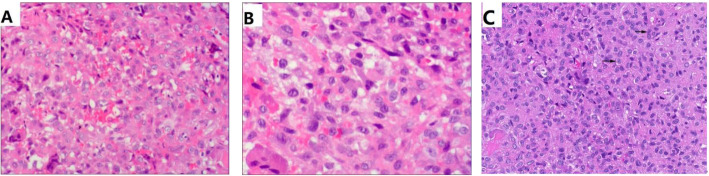
Histological features of Chondroblastoma. (A) Tumor was composed of chondroblasts which were uniform, round to polygonal with well-defined cytoplasmic borders, clear to slightly eosinophilic cytoplasm, and a round to ovoid nucleus (H&E × 100). (B) The tumor cells often had longitudinal grooves and one or more small or inconspicuous nucleoli (H&E × 200). (C) The tumor cells often had longitudinal grooves (black arrow) and one or more small or inconspicuous nucleoli (H&E × 400)

Pathological examination of hematoxylin and eosin (H-E) stained revealed typical polygonal-shaped chondroblasts and osteoclast-like giant cells. The chondroblasts were large and closely packed with a central, characteristically translucent cytoplasm and grooved nucleus (Fig. 3).

**Fig. 4:**
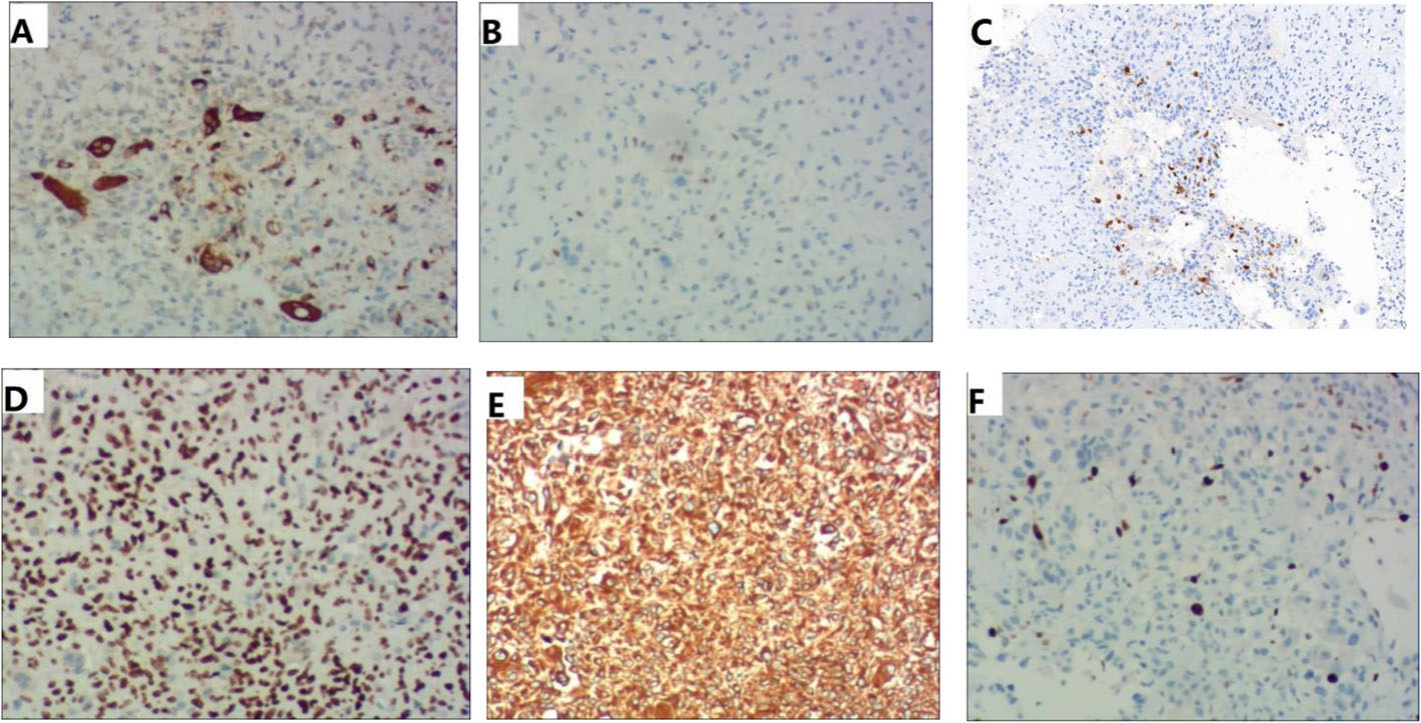
IHC of Chondroblastoma (all staining × 100). (A) The osteoclast-type giant cells were positive for CD68. (B) The chondroblasts were weakly and focally positive for P63. (C) The chondroblasts were focally positive for S100. (D) The chondroblasts were diffusely positive for SATB2. (E) The chondroblasts were diffusely positive for vimentin. (F) The Ki-67 index was approximately 10%

IHC examination made a diagnosis of chondroblastoma (Fig. 4).

**Fig. 5:**
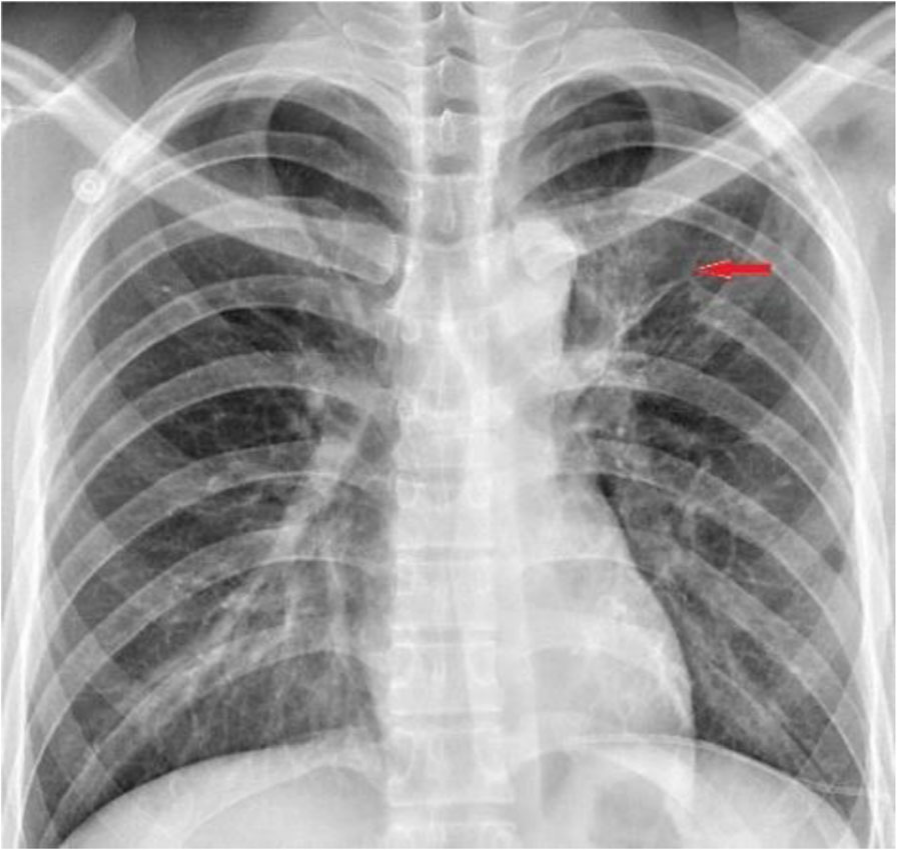
Chest X- ray showed that there was the partial 6th left posterior rib defects and no reconstruction

There were no intraoperative or postoperative complications, and the patient was discharged after 5 days. Patient described only minimal pain, and had only 2 small incisions on the chest wall. There was little impact on lung function, and a shorten chest drainage tube time and hospital stay (Fig. 5).

**Fig. 6:**
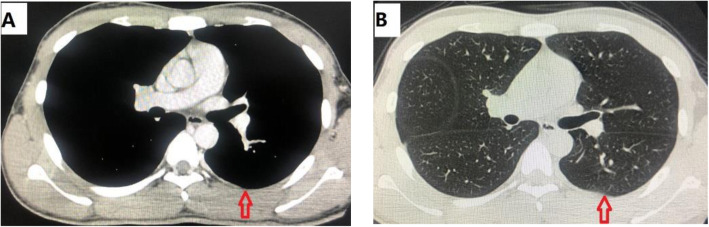
(A) 6 months after surgery; (B) 12 months after surgery. The patient followed up for the second and fourth time and chest CT showed no evidences of recurrence or lung metastases

Radical resection and no adjuvant therapy were given postoperative. The patient was followed every 3 months with chest CT or chest radiograph and blood tests within the first year and every 4 months within the second year. The patient has no chest pain, chest tightness, no abnormal breathing on the chest wall and the lung function is normal (Fig. 6). About 23 months follow-up there was no evidences of recurrence or metastasis.

A timeline showed the whole medical procedure of this case.

## Discussion

Only few chondroblastoma of the rib has been reported in the English literature, there are no definitive or standard treatment guidelines. Rare cases reported that described the diagnosis, radiographic and histological findings, treatment, and follow-up at present.

Local pain is the most common symptom of chondroblastoma. Other signs and symptoms include in swelling, effusion or pathological fractures on rib. Physical examination may show swelling and local tenderness [3, 5]. The patient didn’t describe any pain or discomfort, perhaps because the tumor was small and didn’t invade in adjacent tissues or intercostal nerve.

CT and Magnetic resonance imaging (MRI) are helpful in making the diagnosis [8]. CT may reveal cortical thinning, cortical expansion with erosion, a periosteal reaction, and calcifications in center of the tumor; unusual radiographic changes may also be seen [1] . It can provide the tumor size and identify extension of the lesion into cortical bone, and it is helpful for planning surgery. MRI can identify a soft tissue mass and medullary invasion, and the tumor presents as low signals on T1-weighted images, and with less inhomogeneity and high signals on T2-weighted images. MRI is useful for determining the extent of the tumor, and identifying pathological fractures within the lesions. On contrast-enhanced MRI, the tumors exhibit lobular, marginal, and septal enhancement [9].

Chondroblastoma is relatively benign tumors, and microscopic examination reveals typical polygonal-shaped chondroblasts and osteoclast-like giant cells. H-E shows that chondroblasts are large and closely packed with a central, characteristically translucent cytoplasm and grooved nucleus. In general, chondroblasts and osteoclast-like giant-type cells will be observed [3]. Scattered throughout are small, nucleated giant cells and islands of more mature cartilage. Mitotic features can be present, but are sparse among the generally well-defined nuclei. Calcifications are typically present, and have an intercellular distribution with a “chicken wire” or “picket fence” appearance [2]. The chicken-wire pattern of calcification is helpful in making a firm diagnosis. However, it is not present in the majority of lesions making it a secondary diagnostic criterion. IHC staining for S100 or K36M is positive in chondroblastoma, and is a useful ancillary method for making a diagnosis of chondroblastoma [9, 10].

Chondroblastoma belongs to a benign tumor, but locally aggressive tumor, surgery plays a role in all treatments. Some studies also suggested that chondroblastoma of flat bones are more aggressive than those in long bones [11]. Recurrence after surgery is reported to occur in 10 to 36% of cases [1, 12], and recurrence rates approaching 20% have been reported even with radical resection [5]. In our case we didn’t perform traditional open thoracic surgery instead of VATS resecting the tumor and broken rib. Intraoperative frozen section pathological examination confirmed that the margins of the resected specimen were negative. Compared with the traditional surgery, VATS is minimally invasive and only requires two small incisions, has little or even no impact on lung function, and is associated with a reduced requirement of analgesics, shorter chest drainage tube time, and shorter hospital stay postoperative. In addition, postoperative chest wall numbness is less than that with traditional open thoracic surgery. Earlier reports have advocated curettage for the removal of the tumor [13], but increased recurrence rates have been reported when only curettage is performed for the aggressive tumors [11]. Complete resection is necessary for aggressive tumors (as in our case).

To reduce the risk of recurrence and metastasis, close and regular follow-up is necessary postoperative. Imaging studies are useful for early diagnosis of tumor recurrence. Follow-up can include physical examination and chest CT every 3 months, and bronchoscopy, abdominal ultrasound, brain MRI, and a bone scan every 6 months for the first 3 years is necessary, then every year to 5 years postoperatively [13]. Close and extensive follow-up may improve the outcomes of patients through detection of asymptomatic recurrences and metastasis.

Radiotherapy is a treatment option for patients who are poor surgical candidates, and for patients with recurrent or unresectable disease, and prevent the recurrence, but which is not a standard method treatment [10]. Radiotherapy is not recommended after complete resection, due to the possibility of radiation-induced chondrosarcoma [6]. At present, there is no role for chemotherapy in the management of chondroblastoma [8].

Metastasis most frequently involves the lungs, tends to occur at the time of primary tumor recurrence, and may be present at the time the bone lesion is identified. Lung metastases are clinically non-progressive, and can be treated by limited surgical resection or simple observation.

The primary limitation of this report is the short follow-up time; longer follow-up is necessary to evaluate the efficacy of treatment.

## Conclusions

Chondroblastoma is a benign, locally aggressive tumor that occurs on the rib very rare. The primary treatment is surgical resection for the local lesion. Compared with traditional open thoracic surgery, VATS can achieve the same effect and allows a faster recovery. Radiotherapy is a treatment option for poor surgical candidates, and for patients with recurrent or unresectable disease. Regular follow-up is necessary to identify recurrence and metastases early and provide appropriate treatment.

## Data Availability

Not applicant.

## Abbreviations

CT: Computed tomography
LCH: Langerhans cell histiocytosis
VATS: Video-assisted thoracoscopy surgery
IHC: Immunohistochemical
WHO: World Health Organization
HE staining: Hematoxylin and eosin staining
MRI: Magnetic resonance imaging
